# Research on an Adaptive Method for the Angle Calibration of Roadside LiDAR Point Clouds

**DOI:** 10.3390/s23177542

**Published:** 2023-08-30

**Authors:** Xin Wen, Jiazun Hu, Haiyu Chen, Shichun Huang, Haonan Hu, Hui Zhang

**Affiliations:** School of Intelligent Systems Engineering, Sun Yat-sen University, Shenzhen 518107, China; wenx66@mail2.sysu.edu.cn (X.W.);

**Keywords:** LiDAR, angle calibration, Euler angles, point cloud, Kalman filter, RANSAC, vehicle trajectory, ITS

## Abstract

Light Detection and Ranging (LiDAR), a laser-based technology for environmental perception, finds extensive applications in intelligent transportation. Deployed on roadsides, it provides real-time global traffic data, supporting road safety and research. To overcome accuracy issues arising from sensor misalignment and to facilitate multi-sensor fusion, this paper proposes an adaptive calibration method. The method defines an ideal coordinate system with the road’s forward direction as the X-axis and the intersection line between the vertical plane of the X-axis and the road surface plane as the Y-axis. This method utilizes the Kalman filter (KF) for trajectory smoothing and employs the random sample consensus (RANSAC) algorithm for ground fitting, obtaining the projection of the ideal coordinate system within the LiDAR system coordinate system. By comparing the two coordinate systems and calculating Euler angles, the point cloud is angle-calibrated using rotation matrices. Based on measured data from roadside LiDAR, this paper validates the calibration method. The experimental results demonstrate that the proposed method achieves high precision, with calculated Euler angle errors consistently below 1.7%.

## 1. Introduction

**Figure 1:**
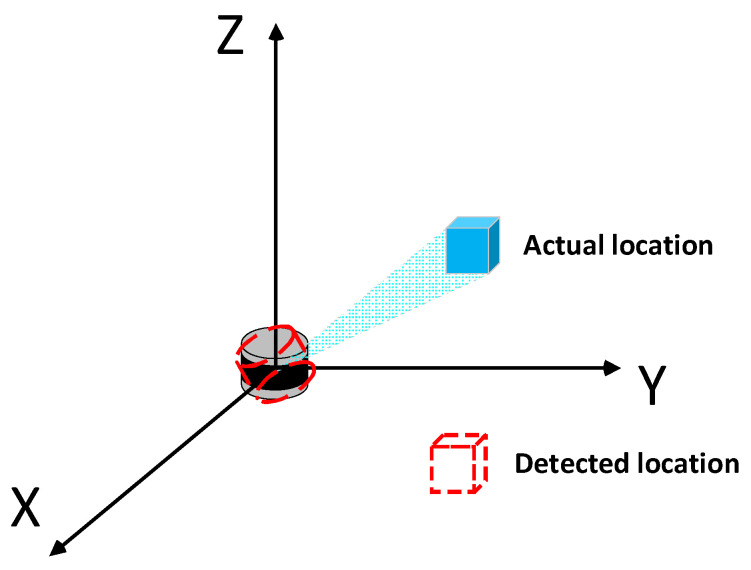
Schematic diagram of LiDAR error detection.

**Figure 2:**
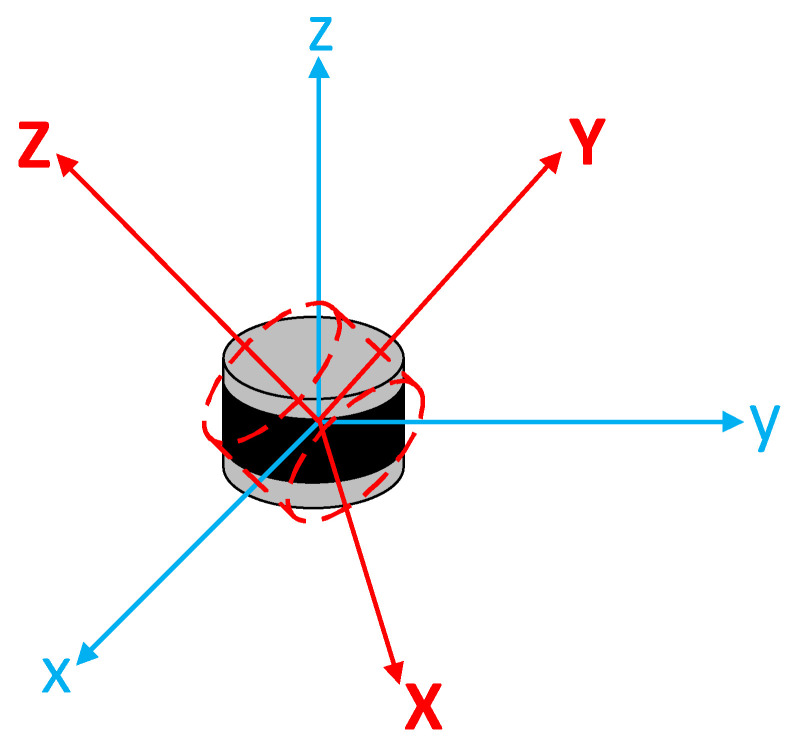
Schematic diagram of the wrong LiDAR mounting angle.

The comprehensive perception and real-time monitoring of traffic information throughout the entire highway network are of great significance for optimizing traffic management strategies and alleviating traffic congestion [1]. To date, traffic information collection and processing technologies have made significant advancements. Among them, the utilization of roadside sensing devices for achieving a holographic perception of traffic information has become a current research hotspot [2]. However, in practical applications, inaccurate vehicle trajectory detection results (Figure 1) are often caused by the suboptimal installation pose of the Light Detection and Ranging (LiDAR, Figure 2). This can also lead to increased difficulties in data analysis and processing during the fusion perception of multiple data sources. Therefore, how to calibrate this offset angle has become a topic worthy of exploration.

**Figure 3:**
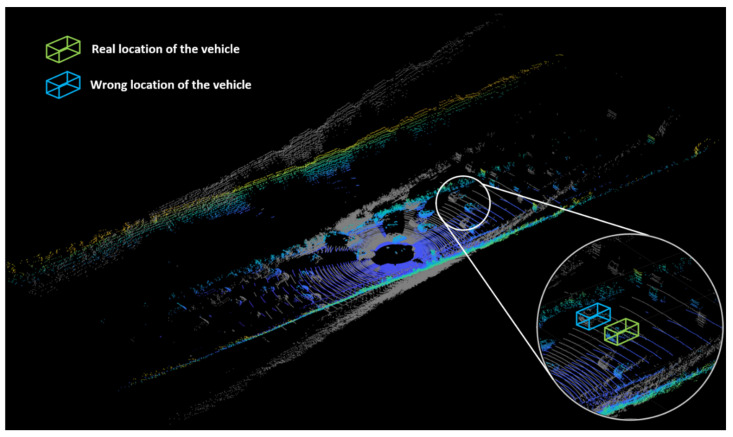
Schematic of the point cloud visualization effect in the case of a normal point cloud and angular deviation (the colored point cloud is the normal case; the gray point cloud is the wrong case).

The commonly used roadside device perception technologies mainly include sensing technologies composed of devices such as Light Detection and Ranging (LiDAR) [3,4], Millimeter Wave Radar (MMW-Radar) [5,6], inductive sensors [7], cameras [8,9], etc. These sensing technologies can be used to monitor and collect real-time data on the movement of vehicles within a certain range on the road surface through either single-sensor perception or multi-sensor fusion perception. Based on this, comprehensive and accurate data on traffic conditions throughout the entire road segment can be obtained. Wang [10] summarized the advantages, disadvantages, and detection ranges of the aforementioned perception devices, as shown in Table 1. LiDAR is characterized by high accuracy, high resolution, and a wide perception range [11], enabling a more precise perception and measurement of vehicle trajectories. When placed at elevated points in a traffic scene, it allows for an overhead view to observe the overall situation, enabling real-time perception of the high-precision motion trajectories of each object in the traffic scene. However, in practical applications, inaccuracies in the installation angle of LiDAR or minor movements that occur during the installation process can lead to deviations in the scanning results of the LiDAR, as shown in Figure 3 (the point cloud data sourced from the KITTI dataset [12]). These factors can have a significant impact on perceiving the trajectories of vehicles, primarily manifested as follows:

1. Positioning deviation: Angle deviations in LiDAR detection can lead to spatial deviations in vehicle positions, resulting in a decrease in the positioning accuracy of vehicle trajectories.
2. Trajectory drift: Angle deviations can cause an amplification of the offset error between the LiDAR-perceived vehicle trajectory and the actual trajectory, leading to the presence of trajectory drift.
3. Trajectory shape changes: Due to the presence of angle deviations, the perceived shape of the vehicle trajectory may undergo changes, potentially causing errors in determining the vehicle’s driving state and impacting subsequent traffic information processing and analysis.
4. Negative impact on multi-sensor fusion perception: Angle deviations in LiDAR can result in inconsistencies between the perceived data and the data obtained from other sensors such as cameras and radars. Such inconsistencies make it challenging to accurately integrate and process data in a multi-sensor fusion perception system, affecting the comprehensive understanding and holistic analysis of traffic situations.

The angle calibration of LiDAR point clouds refers to adjusting the collected point cloud data’s posture or rotation to align or align them with a specific reference coordinate system. To date, a significant amount of research has been conducted by domestic and international experts and scholars on angle calibration methods for LiDAR point clouds. These methods can be roughly classified into two categories: those with fixed reference objects and those with non-fixed reference objects. Methods with fixed reference objects primarily involve identifying known reference objects’ shape, position, and other information to calculate the LiDAR’s angular deviation, achieving the purpose of calibration. For example, Y. Park [13] proposed the use of a polygon plane identification board, Z. Pusztai [14] introduced a standard square marker board, and Zhu [15] employed a checkerboard marker board for calibration. However, these methods still have certain limitations for the angle calibration of roadside LiDAR point clouds. Operators need to set up reference markers for angle calibration, which not only requires significant effort but also poses safety concerns for the operators. Methods with non-fixed reference objects can be classified into two types: shape-based calibration and motion-based calibration [16]. For example, Y. Shen [17] proposed using detected object contours as references, and P. Moghadam [18] introduced a shape-based calibration method based on line features from depth sensors and cameras. These methods belong to shape-based calibration. Additionally, Z. Taylor [19] proposed the use of hand–eye calibration for the external parameter calibration of multiple sensors, which falls under motion-based calibration. These methods effectively calibrate and accurately position the geometric relationship between LiDAR and cameras or other sensing devices. However, they are not suitable for the angle calibration of individual LiDAR point cloud. Additionally, to facilitate the subsequent processing and analysis of point cloud segmentation and trajectory data, researchers prefer the detected road point clouds to be parallel to a specific coordinate plane in the LiDAR system’s coordinate system, while the direction of the road should align with a particular coordinate axis.

In the field of intelligent transportation, point clouds registration [20,21] is a common method used to merge different LiDAR point cloud data and perform angle calibration. Its purpose is to align point cloud data from different LiDAR sensors into a shared coordinate system. By utilizing feature point matching algorithms, corresponding point pairs between different point clouds are identified, and the pose transformation (such as translation and rotation) between them is calculated to calibrate the coordinate systems of different LiDAR sensors. This enables precise data fusion and analysis. Zhang [22] proposed a three-dimensional registration method based on prior knowledge of traffic signs and traffic scenes. Y. Rui [23] proposed a method for registering data from multiple roadside LiDAR sensors based on point cloud features. These methods have shown remarkable performance in addressing the fusion and registration problem of multiple LiDAR information. However, when it comes to fusing vehicle trajectory information from different types of roadside sensing devices, determining a universally applicable coordinate system becomes crucial for achieving data fusion.

Based on the above, this paper proposes a method for calibrating the angle deviation of roadside LiDAR point clouds. Unlike traditional calibration methods, this method does not rely on fixed reference objects but instead utilizes traffic elements (road heading direction and ground surface) as reference objects. By establishing an ideal coordinate system, the road heading direction is defined as the X-axis direction, and the direction of the intersection line perpendicular to the X-axis plane and the road surface plane is chosen as the Y-axis direction, thus constructing a reference framework adapted to the road. In this ideal coordinate system, the *XY* plane is essentially parallel to the road surface. This method primarily utilizes the Kalman filter (KF) to improve the smoothness of the trajectory. Afterward, based on a certain number of vehicle trajectory points and the fitted road surface plane, it calculates the projection of the ideal coordinate system *XYZ* onto the LiDAR system’s coordinate system *xyz*. In this projection, the road heading direction is taken as the X-axis direction, and the direction of the intersection line perpendicular to the X-axis plane and the road surface plane is taken as the Y-axis direction. Finally, the Euler angles are computed to achieve point cloud calibration using rotation matrices.

The primary contributions of this article can be summarized as follows:

- Adaptive calibration method: This paper proposes a simple and efficient adaptive calibration method to address the accuracy issues of LiDAR sensors in roadside intelligent perception systems. By leveraging the Kalman filter (KF) and the random sample consensus (RANSAC) algorithm, this method ensures real-time calibration without the need for manual intervention.
- Ideal coordinate system definition: The method defines an ideal coordinate system based on the road’s forward direction as the X-axis and the intersection line between the vertical plane of the X-axis and the road surface plane as the Y-axis. This ideal coordinate system serves as a reference for calibration, enhancing the precision and stability of the calibration process. Additionally, it provides a universally applicable reference for further multi-sensor fusion perception.

As mentioned earlier, research on methods for obtaining vehicle trajectories using LiDAR is already quite mature. Therefore, this paper does not provide a further description of vehicle trajectory perception methods using LiDAR. Instead, it primarily focuses on validating the feasibility of the calibration method based on the analysis of vehicle trajectory data derived from measured roadside point cloud data. The results demonstrate that the calculated Euler angles have an error rate consistently below 1.7%, indicating extremely high precision.

The rest of the paper Is organized as follows: Section 2 provides a detailed introduction to the basic principles and working mechanism of the proposed point cloud calibration method. In Section 3, comprehensive analysis and experimental validation are conducted using the measured data to assess the feasibility and effectiveness of the calibration method. Finally, in Section 4, the summary and discussion section, the experimental results are summarized, conclusions are drawn, and future research prospects are outlined.

## 2. The Basic Principles of Point Cloud Calibration Method

**Figure 4:**
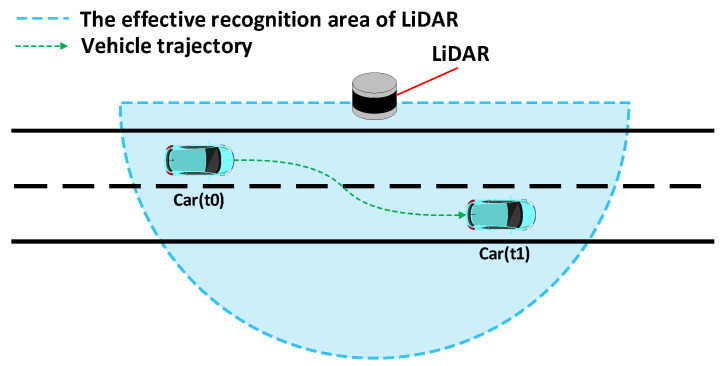
Schematic diagram of the application scenario.

**Figure 5:**
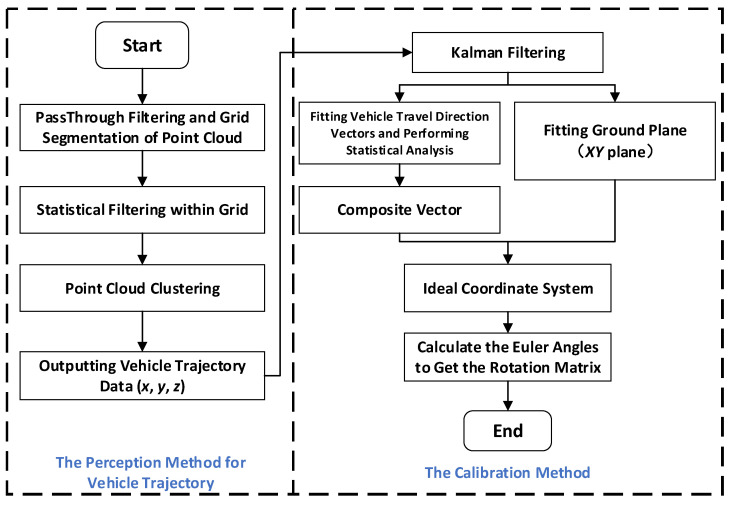
Flow chart of the point cloud calibration algorithm.

The paper presents a calibration method for addressing angle deviations in roadside LiDAR. Its application scenario is depicted in Figure 4, utilizing traffic elements (road heading direction and ground surface) as reference objects. The objective is to improve the accuracy of vehicle position detection and facilitate subsequent data processing and analysis. This section provides a detailed introduction to the basic principles of this calibration method, with Figure 5 illustrating the basic algorithm flow of the point cloud calibration method. As the methods for vehicle trajectory perception using LiDAR are already well developed, this section primarily focuses on introducing the calibration method as the main content. 

### 2.1. Statistical Analysis of Vehicle Driving Directions

**Figure 6:**
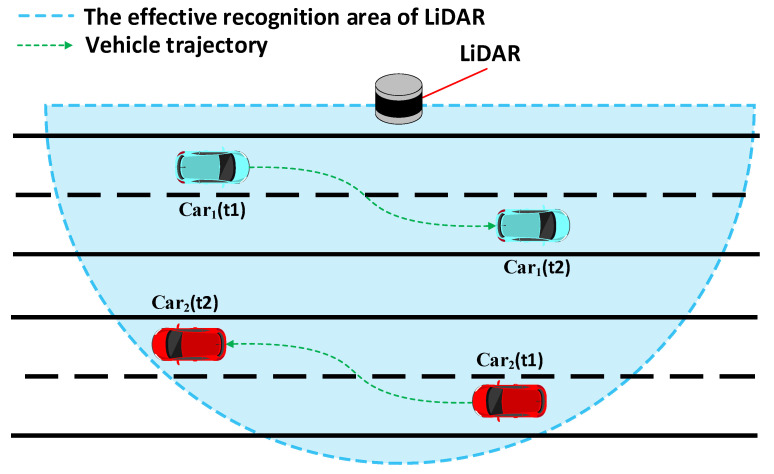
Schematic diagram of the experimental scenario for the statistical analysis of vehicle travel directions.

**Figure 7:**
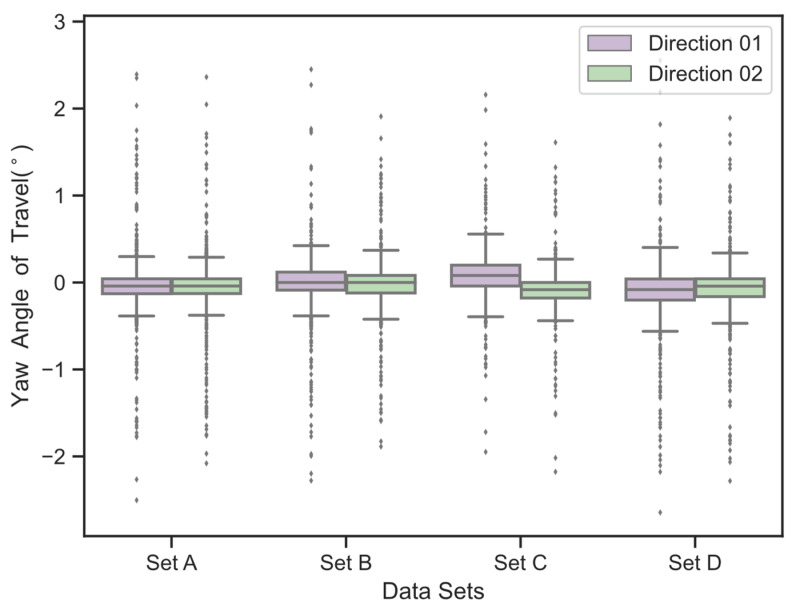
Boxplot: statistical analysis of the heading angles (in degrees) in four vehicle trajectory data sets.

**Figure 8:**
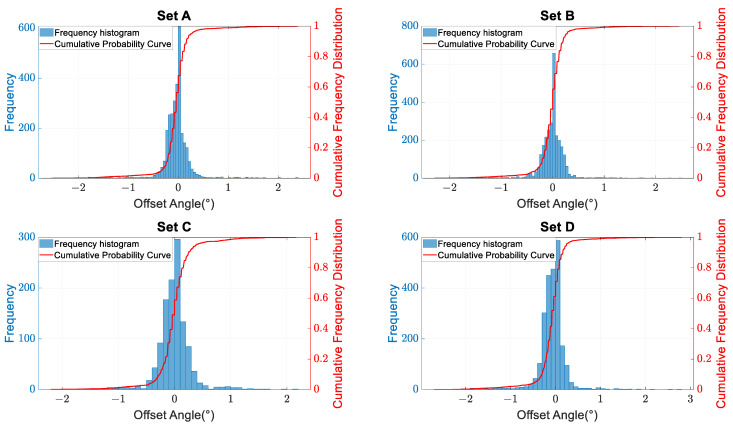
Statistical analysis of probability distribution: the angle (in degrees) between the vehicle’s driving direction and the forward direction of the road.

The vehicle’s driving direction, based on LiDAR perception, is a crucial reference for calculating the projection of the road’s forward direction onto the coordinate system of the LiDAR system. This paper presents a statistical analysis of the angles between the perceived vehicle driving directions and the road heading directions based on four sets of measured LiDAR data. The LiDAR used in the experiments has been calibrated for angles using fixed reference markers, aligning the X-axis of the system’s coordinate system parallel to the road heading direction. The perception range was set as a 12 m segment, with 6 m in front and 6 m behind the LiDAR, as depicted in Figure 6. The statistical results are shown in Figure 7 and Figure 8.

The experimental results indicate that the expected value of the angle between the vehicle’s driving direction and the road heading direction is close to zero. Therefore, theoretically, it is possible to estimate the road heading direction based on a certain amount of vehicle trajectory data.

### 2.2. Smoothing of Vehicle Trajectories

The Kalman filter (KF) is an efficient recursive filter, also known as an autoregressive filter, which is capable of estimating the state of a dynamic system from a series of incomplete and noisy measurements [24]. Due to the detection distance and occlusion issues, vehicle trajectory data based on LiDAR perception may suffer from false detections. The purpose of using the KF is to optimize the vehicle trajectories and improve their smoothness, making them closer to the true trajectory data. Compared to other trajectory smoothing algorithms, the KF exhibits a series of distinct advantages, which is the primary reason for selecting the KF in this paper. The following are the main advantages of the KF:

- Adaptability: The KF demonstrates outstanding adaptability to trajectory smoothing problems in linear systems when the data satisfy the conditions of linearity and Gaussian distribution. Under such conditions, the KF provides accurate and stable estimation results.
- Low computational cost: The KF exhibits relatively low computational complexity. By processing data using a recursive approach, it does not require a substantial amount of computational resources, making it suitable for real-time applications or scenarios with limited computational capacity.
- Optimality: Under the assumptions of linearity and Gaussian distribution, the KF represents the optimal solution for minimum mean square error estimation. This implies that under ideal conditions, it can achieve the best trajectory smoothing effect and deliver the most accurate estimation results.
- Low storage requirement: The KF only necessitates storing the current state and covariance information, without the need to retain historical data. Therefore, it places relatively low demands on storage resources, making it advantageous for applications in resource-constrained environments, such as embedded systems.

In the KF, the state transition and measurement models are expressed as:

(1) $${\hat{x}}_{k}=A{\hat{x}}_{k-1}+Bu_{k}+W_{k}$$

(2) $$z_{k}=H{\hat{x}}_{k}+V_{k}$$

where ${\hat{x}}_{k}$ is the estimate of the prior state at time k, $u_{k}$ is the control vector at time k, and $W_{k}$ is the process noise at time k, which follows a Gaussian distribution with mean 0 and covariance denoted as $Q_{k}$. $z_{k}$ is the observed value at time k, which is the detected vehicle position and is a known value, and $V_{k}$ is the measurement noise at time k, which also follows a Gaussian distribution with mean 0 and covariance denoted as $R_{k}$. A is the state transition matrix that maps the state at time k−1 to the state at time k, B is the control input matrix that maps the state at time k−1 to the control input at time k, and H is the transformation matrix that maps the state to the measurement space.

If the observed value $z_{k}$ is known, then the true state B of the estimated system can be obtained. The prediction process can be represented as follows:

(3) $${\hat{x}}_{k|k-1}=A{\hat{x}}_{k-1|k-1}+Bu_{k}$$

(4) $$P_{k|k-1}=AP_{k-1|k-1}A^{T}+Q_{k}$$

The filtering process can be represented as follows:

(5) $$K_{k}=P_{k|k-1}H^{T}\cdot {\left(HP_{k|k-1}H^{T}+R_{k}\right)}^{-1}$$

(6) $${\hat{x}}_{k|k}={\hat{x}}_{k|k-1}+K_{k}\left(z_{k}-H{\hat{x}}_{k|k-1}\right)$$

(7) $$P_{k|k}=\left(I-K_{k}H\right)P_{k|k-1}$$

where $K_{k}$ is the Kalman gain at time k. $P_{k|k-1}$ and $P_{k|k}$ are the covariance at time k and the filtered covariance at time k, respectively, obtained based on the state at time k−1. Their initial values can be any non-zero number.${\hat{x}}_{k|k-1}$ and ${\hat{x}}_{k|k}$ are the state prediction at time k and the filtered state at time k (i.e., the final predicted state ${\hat{x}}_{k}$), obtained based on the state at time k−1, respectively. They can have any initial values. I is the identity matrix.

**Figure 9:**
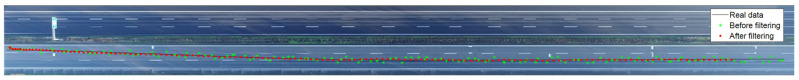
Schematic diagram of the effect of the KF algorithm on the smoothing of vehicle trajectory points.

Taking the driving trajectory of a vehicle with lane-changing behavior from the highD dataset [25] as an example, the effectiveness of the KF was tested by introducing some noise, as shown in Figure 9. It can be observed that the trajectory was optimized to a certain extent. Although the test was conducted using two-dimensional vehicle trajectories, the KF can also be applied to optimize three-dimensional vehicle driving trajectories [26].

### 2.3. The Principles of Fitting the Ideal Coordinate System

Based on statistical analysis of vehicle trajectory and the RANSAC algorithm, this paper determines the projection of the ideal coordinate system in the coordinate system of the LiDAR system. First, by performing a statistical analysis of the vehicle trajectory data, it is possible to fit the X-axis direction of the ideal coordinate system. Next, the RANSAC algorithm is utilized to fit the ground and find the intersection between the plane perpendicular to the X-axis and the ground, which serves as the Y-axis direction of the ideal coordinate system. Finally, the cross-product operation between the X-axis and Y-axis is performed to determine the Z-axis direction of the ideal coordinate system. The design of this process ensures that the three coordinate axes of the ideal coordinate system are mutually orthogonal.

#### 2.3.1. Fitting the X-Axis

This paper utilizes the accumulated direction vectors of vehicle trajectory to calculate the direction of the X-axis of the ideal coordinate system in the coordinate system of the LiDAR system. The algorithm flow for fitting the X-axis can be summarized into the following four steps:

1. Preprocessing and data preparation: The original data are preprocessed to filter the vehicle trajectory data within the region of interest.
2. Calculating the direction of travel vector for each vehicle: Calculate the motion direction vector for each vehicle and record it.
3. Statistical analysis of travel direction vectors: Perform statistical analysis on the computed vehicle motion direction vectors to determine the direction of road advancement in the coordinate system.
4. Evaluation and validation: Validate the analysis results to determine if there is a significant deviation from the ideal road advancement direction.

Equation (8) represents the formula for vector summation.

(8) $$v_{X}=\frac{\left[v_{1}+v_{2}+\ldots +v_{n}\right]}{\left\|\left[v_{1}+v_{2}+\ldots +v_{n}\right]\right\|}=\frac{\left[x_{1}+x_{2}+\ldots +x_{n},y_{1}+y_{2}+\ldots +y_{n},z_{1}+z_{2}+\ldots +z_{n}\right]}{\left\|\left[x_{1}+x_{2}+\ldots +x_{n},y_{1}+y_{2}+\ldots +y_{n},z_{1}+z_{2}+\ldots +z_{n}\right]\right\|}$$

where $v_{X}=[a_{X},b_{X},c_{X}]$ is the sum vector (normalized) indicating the direction of the X-axis. $v_{1},v_{2},\ldots ,v_{n}$ are the normalized direction vectors of a vehicle’s motion, where $v_{i}=[x_{i},y_{i},z_{i}]$.

#### 2.3.2. Fitting the Y-Axis

This paper considers the direction of the intersection between the X-axis vertical plane and the ground plane as the Y-axis direction. The main approach is based on implementing the RANSAC algorithm for fitting the ground plane. Random sample consensus (RANSAC) [27] is an iterative method used to estimate mathematical model parameters based on a set of observation data containing outliers. RANSAC is a non-deterministic algorithm as it produces reasonable results with a certain probability, and this probability increases with the number of allowed iterations. The method is commonly employed for fitting plane equations. Here are the advantages of the RANSAC algorithm compared to other plane fitting algorithms:

- Robustness: RANSAC is capable of handling data sets containing outliers and noise, as it can ignore these abnormal values and produce better fitting results.
- No dependency on initial values: RANSAC does not require initial values or prior information. It obtains an initial fitting model through random sampling, eliminating the need for preliminary estimations.
- Simplicity and efficiency: The basic idea of the RANSAC algorithm is straightforward, making it easy to understand and implement. By randomly selecting samples and iterating to find the best model, it exhibits high computational efficiency in most cases.
- Adjustable parameters: RANSAC allows setting parameters to control the number of samples and the fitting threshold, enabling fine-tuning according to specific problem requirements and achieving improved fitting results.

The algorithm flow for fitting a plane using the RANSAC algorithm is as follows:

1. Set the maximum number of iterations, max_iterations.
2. Set the distance threshold, threshold, for determining inliers.
3. Initialize the parameters for the best-fitting plane: best_plane=None and best_inliers are set as an empty set, best_num_inliers=0.
4. Iterate max_iterations times:
  a. Randomly select the smallest set of points from the input data.
  b. Use the selected points to estimate plane parameters (e.g., through least-squares fitting).
  c. By calculating the distance between each point and the estimated plane, identify the inliers.
  d. Calculate the number of inliers with distances below threshold.
  e. If the number of inliers is greater than best_num_inliers, update best_num_inliers, best_inliers, and best_plane.
5. Re-fit the plane using all inliers from step 4(e) to obtain the final estimated plane parameters.
6. The final result, A, represents the plane obtained via fitting using the RANSAC algorithm.

Due to the possibility that best_plane may not be parallel to the X-axis, the intersection between best_plane and $P_{x}$, which is perpendicular to the X-axis, is taken as the direction of the Y-axis. The calculation process for the direction vector of the Y-axis is as follows:

1. Determine the normal vectors of the two planes: The normal vector of best_plane is $n_{{}_{G}}=\left[x_{g},{\;y}_{g},{\;z}_{g}\right]$. The normal vector of plane $P_{x}$ is $n_{P}=\left[x_{p},{\;y}_{p},{\;z}_{p}\right]$.
2. Calculate the direction vector of the intersection, $n_{n}$:
  (9) $$n_{n}=n_{G}\times n_{P}=\left[x_{n},{\;y}_{n},{\;z}_{n}\right]=\left[y_{g}∙z_{p}-z_{g}∙y_{p},z_{g}∙x_{p}-x_{g}∙z_{p},x_{g}∙y_{p}-y_{g}∙x_{p}\right]$$
3. Normalize the direction vector of the intersection: The magnitude $\left\|n_{n}\right\|$ of the intersection’s direction vector can be calculated using Equation (10):
  (10) $$\left\|n_{n}\right\|=\sqrt{x_{n}^{2}{+y}_{n}^{2}{+z}_{n}^{2}}$$

The normalized $v_{Y}=n_{n}/\left\|n_{n}\right\|=[a_{Y},b_{Y},c_{Y}]$ represents the direction of the Y-axis.

#### 2.3.3. Fitting the Z-Axis

The direction of the Z-axis, $v_{Z}=[a_{Z},b_{Z},c_{Z}]$, can be obtained through the cross-product operation of the X-axis direction vector $v_{X}$ and the Y-axis direction vector $v_{Y}$, and the calculation process can be represented using Equation (11):

(11) $$v_{Z}=v_{X}\times v_{Y}=[a_{X},b_{X},c_{X}]\times [a_{Y},b_{Y},c_{Y}]=\left[b_{X}c_{Y}-c_{X}b_{Y},c_{X}a_{Y}-a_{X}c_{Y},a_{X}b_{Y}-b_{X}a_{Y}\right]$$

### 2.4. Principles of Point Cloud Angle Calibration

This paper employs rotation matrices to achieve angle calibration of LiDAR point clouds. A mathematical relationship exists between Euler angles and rotation matrices, where Euler angles are a method of describing the rotational orientation of objects in three-dimensional space using three consecutive angles of rotation. The rotation matrix [28] is a linear transformation matrix used to convert the rotational operations described by Euler angles into matrix multiplication, enabling the rotation transformation of three-dimensional objects. In this subsection, the LiDAR point cloud is treated as a three-dimensional object, and angle calibration of the point cloud is achieved by calculating Euler angles to obtain the rotation matrix.

#### 2.4.1. Euler Angle

**Figure 10:**
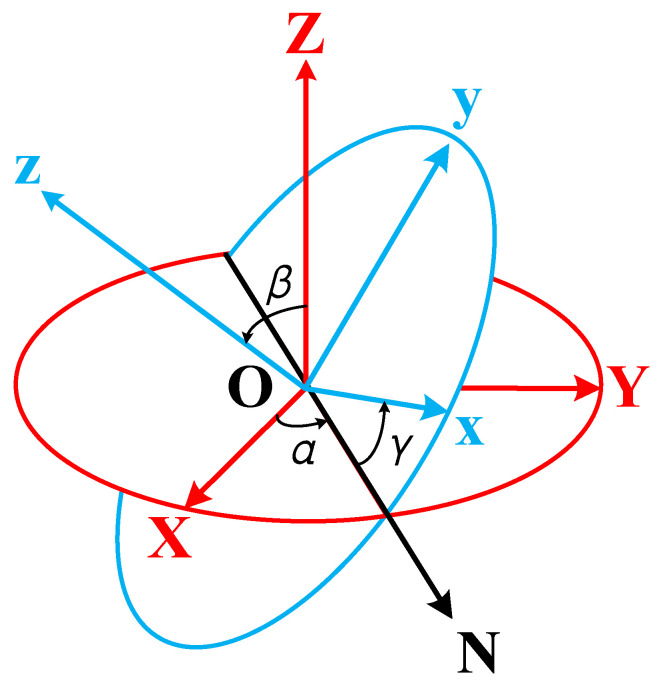
Three Euler angles: (α,β,γ). The red axes represent the *XYZ* axes, and the blue axes represent the *xyz* axes. The black line is the intersection line (*N*).

Euler angles are used to describe the rotational orientation of an object in three-dimensional Euclidean space [29]. They consist of three rotation angles, typically denoted by symbols (α,β,γ). Each angle represents the amount of rotation around a specific axis. Referring to Figure 10, *xyz* represents the reference axes of the reference frame, and *XYZ* represents the object’s own coordinate axes. The intersection line of the *xy* plane and the *XY* plane is denoted by *N*. For a *zxz* intrinsic Euler angle convention, the angles can be statically defined as follows: α (precession angle) is the angle between the X-axis and *N*, β (nutation angle) is the angle between the z-axis and Z-axis, and γ (spin angle) is the angle between *N* and the x-axis. The reference frame composed of *xyz* (fixed frame) is also known as the laboratory reference frame, which remains stationary. On the other hand, the coordinate system composed of *XYZ* (body-fixed frame) is fixed to the object and rotates with the object during its rotation.

Regarding the order and labeling of angles, as well as the specification of the two axes of the angle, there are no conventions. Therefore, when using Euler angles, it is essential to explicitly define the rotation angles and rotation axes. In practical applications, different proper Euler angles can be used to describe the rotation of an object.

#### 2.4.2. Rotation Matrix

This paper utilizes rotation matrices to achieve the angle calibration of point cloud data. The specified rotation matrix is composed of three elementary rotation matrices, as shown in Equation (12):

(12) $$[R]=\left[\begin{array}{ccc} \cos\gamma & \sin\gamma & 0\\ -\sin\gamma & \cos\gamma & 0\\ 0 & 0 & 1 \end{array}\right]\left[\begin{array}{ccc} 1 & 0 & 0\\ 0 & \cos\beta & \sin\beta \\ 0 & -\sin\beta & \cos\beta \end{array}\right]\left[\begin{array}{ccc} \cos\alpha & \sin\alpha & 0\\ -\sin\alpha & \cos\alpha & 0\\ 0 & 0 & 1 \end{array}\right]$$

In the equation, from right to left, it represents rotations around the Z-axis (α), the intersection line (β), and the z-axis (γ). After some calculations, we obtain:

(13) $$[R]=\left[\begin{array}{ccc} \cos\alpha \cos\gamma -\cos\beta \sin\alpha \sin\gamma & \sin\alpha \cos\gamma +\cos\beta \cos\alpha \sin\gamma & \sin\beta \sin\gamma \\ -\cos\alpha \sin\gamma -\cos\beta \sin\alpha \cos\gamma & -\sin\alpha \sin\gamma +\cos\beta \cos\alpha \cos\gamma & \sin\beta \cos\gamma \\ \sin\beta \sin\alpha & -\sin\beta \cos\alpha & \cos\beta \end{array}\right]$$

## 3. Experiments

In the experiment, researchers selected a mechanical LiDAR (Light Detection and Ranging) system with 64 channels and the capability of performing three-dimensional high-speed scanning. The LiDAR’s scanning frequency is 10 Hz, with a horizontal resolution of 0.09°, a vertical field of view of 26.8°, a distance accuracy of 2 cm, and a maximum detectable range of 120 m.

### 3.1. Experimental Procedure

**Figure 11:**
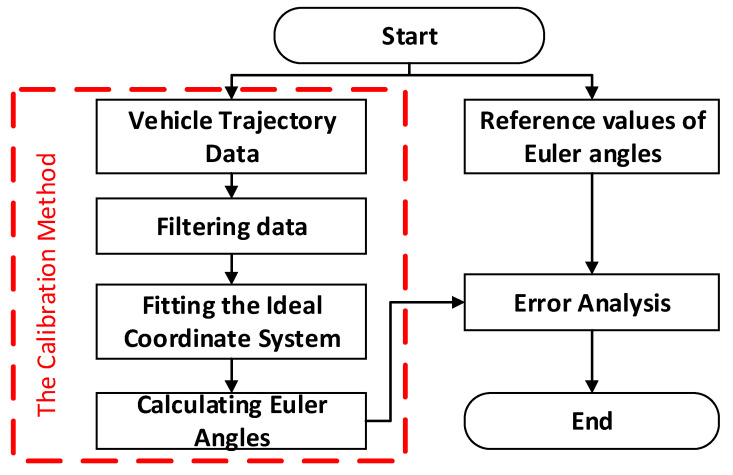
Verification algorithm flowchart for the LiDAR angle calibration method.

The roadside perception system in the experiment involved mounting an uncalibrated LiDAR system at a height of 3 m on the side of a highway. The system continuously collected data for approximately 15 min, capturing trajectory information for nearly 1200 vehicles. Subsequently, the researchers used fixed reference markers for measurements to calculate the offset angle of the LiDAR system. This allowed them to determine the transformation relationship between the LiDAR system coordinate system and the ideal coordinate system, expressed as *zxz* Euler angles: α=4°, β=10°, and γ=6°. These data were used to validate the accuracy of the proposed LiDAR point cloud angle calibration method. To provide a clear description of the validation process, this paper includes a validation algorithm flowchart for the LiDAR angle calibration method, as shown in Figure 11. The flowchart illustrates the steps and computations involved in the validation process, ensuring the accuracy of the calibration method.

### 3.2. Experimental Results and Analysis

**Figure 12:**
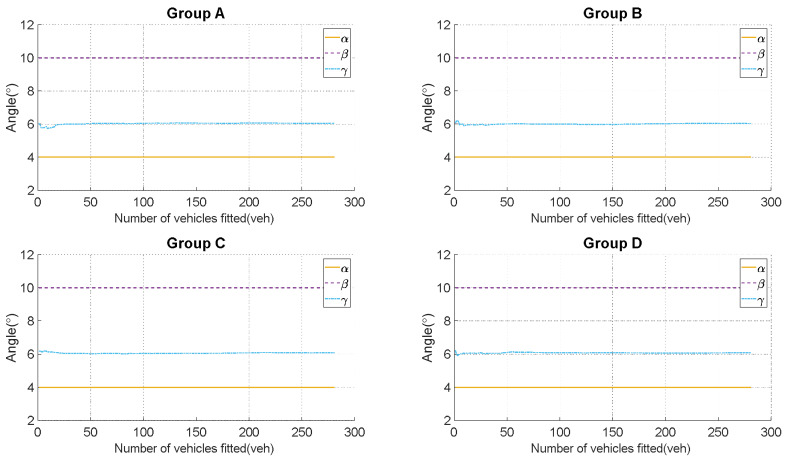
Calculated results of (α,β,γ): Complete set of calculation results for the four experimental groups, with approximately 280 vehicles fitted in each group.

This paper validates the proposed calibration method for the LiDAR system. Based on the collected vehicle trajectory data, vehicle trajectories within a 10-m range before and after the LiDAR deployment locations are selected. The trajectory data is categorized into four experimental groups based on the vehicle IDs, with lower ID numbers being assigned to vehicles that entered the recording area earlier. These experimental groups are specifically labeled as Group A, Group B, Group C, and Group D in accordance with academic conventions. The partial calculation results for (α,β,γ) are shown in Table 2, and the summarized results of all calculations are presented in Figure 12.

**Figure 13:**
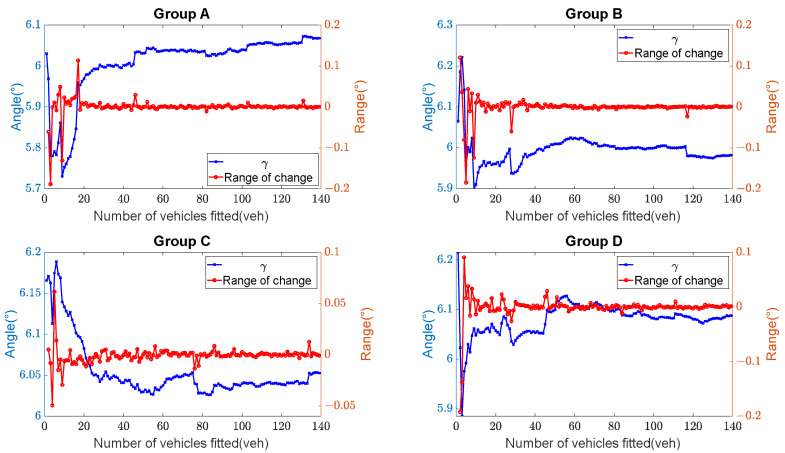
Calculated results of the calibration angle (angle γ): Temporal evolution and amplitude analysis of angle γ.

The experimental results indicate that there is almost no error between the calculated values of α and β (their reference values: α=4° and β=10°). However, for γ (reference value: γ=6°), there is a relatively larger error. The relationship between the fitted vehicle trajectory count and the calculated result of γ, as well as its relationship with the amplitude variation, are shown in Figure 13. It can be observed that when the number of fitted vehicles exceeds 50, the calculated results for γ stabilize, and the error rate for γ remains below 1.7%. This demonstrates that the method possesses a high level of reliability and accuracy in addressing the calibration issues of LiDAR point clouds.

## 4. Discussion

In typical scenarios, when deploying a LiDAR within a roadside environment, factors such as the minor vibrations induced during the LiDAR’s operation, suboptimal mounting angles, and vibrations caused by high-speed vehicle passages can collectively result in deviations between the LiDAR’s scanning angles and the ideal scenario. Such deviations in scanning angles may potentially undermine the efficacy of the roadside LiDAR perception system, subsequently increasing the intricacy of post-data processing and analysis. Therefore, the investigation of methods for LiDAR angle deviation calibration holds utmost significance.

This paper presents a calibration method suitable for roadside LiDAR systems, the essence of which lies in establishing an ideal coordinate system and leveraging traffic elements (including the ground and vehicle trajectories) as reference benchmarks. Calibration is achieved through precise processing of LiDAR point cloud data. The feasibility of this method is validated through a comprehensive experimental design. The experimental results unequivocally demonstrate that this method has achieved remarkable effectiveness in addressing the angle calibration challenges of roadside LiDAR systems. In comparison to conventional calibration methods based on a fixed reference board [13,14,15], this approach offers the following advantages:

- Cost and resource savings: In remote urban areas, embedding the program into the perception system is sufficient to achieve adaptive calibration. Furthermore, it can automatically calibrate the angle deviation of LiDAR periodically, ensuring the perception system maintains high-precision sensing capabilities.
- Applicability to multi-LiDAR systems: In scenarios with a linear layout of the perception area and the presence of multiple LiDAR sensors, relying on known LiDAR deployment positions greatly simplifies the challenges of fusing data from multiple sources, thereby enhancing the accuracy of fused perception results.

However, in reality, the method of using fixed reference boards still holds a prominent position in terms of accuracy. Researchers’ analyses reveal that the crucial factor affecting the calibration accuracy of the method lies in the vehicle trajectory detection algorithm. Future research efforts will be concentrated on optimizing this aspect. The method proposed in [22] suggests using traffic signs as calibration references, essentially applying the fixed reference boards approach to roadside environments. Nevertheless, in certain road scenarios, available traffic signs might not always be present, limiting the applicability of this method. In [23], point cloud features are employed as references, and the registration of point clouds is achieved through optimization algorithms. Methods [22,23] are better suited for registering and fusing point clouds from multiple LiDAR sensors, yet they do not adequately address the issue of LiDAR angle deviations. Consequently, these methods fail to effectively enhance the precision of vehicle trajectory recognition.

In summary, addressing the issue of LiDAR angle deviations in roadside LiDAR perception systems, the proposed method in this paper offers a sound solution to this problem, while also streamlining the challenges of multi- LiDAR data fusion and registration. The primary advantages of this method can be summarized as follows:

- Simplified parameter configuration: As an adaptive calibration method, it requires only the specification of the vehicle trajectory data for fitting, eliminating the need for intricate parameter tuning.
- Cost-effectiveness: It significantly saves human and material resources.
- High safety: It enhances operational safety by eliminating the need for manual intervention by operators at the roadside.
- Non-interference with normal operations: Embedded within the perception system’s program, it does not disrupt the normal sensing functionality of the LiDAR system, accurately capturing vehicle information and trajectory data.
- Adaptation to multi-LiDAR fusion perception: For continuous placement of multiple LiDAR sensors along long straight road sections, this calibration method simplifies the complexity of multi-source data fusion perception by requiring only the determination of LiDAR deployment positions.
- Enhancing trajectory data accuracy: By addressing the issue of angular deviation, this approach contributes to the improved perception accuracy of vehicle trajectory data. Consequently, it establishes a more precise foundational dataset for subsequent applications.

The limitations of this method can be summarized as follows:

- Initial instability: During the initial operational phase of the system, when vehicles exhibit behaviors like lane changes within the region of interest, the limited quantity of fitted vehicle trajectory data might lead to reduced perception accuracy. However, as the number of fitted vehicle trajectories increases, accuracy gradually improves.
- Real-time impact: In the early stages of system operation, performing the calibration algorithm for almost every perceived vehicle trajectory data point might affect the real-time nature of the perception system, resulting in increased algorithm execution times.
- Applicability to complex road segments: This calibration method performs optimally on straight road segments; however, it might be challenging to find suitable road segments for calibration in complex road scenarios.

Future research will extensively explore the application of the calibration algorithm to handle complex road segments with varying curvatures. Simultaneously, efforts will be directed toward enhancing algorithm efficiency and robustness.

## 5. Conclusions

This paper introduces an innovative LiDAR angle calibration method that eliminates the need for a calibration board, effectively tackling the problem of point cloud angle deviation caused by the suboptimal installation poses of roadside LiDAR systems. The proposed method relies solely on perception targets (vehicle trajectories and the ground) for calibration. This paper validates the feasibility of the proposed calibration method based on real-world roadside data. The experimental results demonstrate that when the number of fitted vehicle trajectories exceeds 50, the calculated Euler angles remain relatively stable, with an error rate of less than 1.7%. These experimental results fully demonstrate that the proposed calibration method in this paper possesses extremely high accuracy and reliability.

During the initialization phase of roadside perception systems, this method can be utilized to achieve adaptive calibration of point cloud angles. Notably, this approach is robust to the influence of road slopes. By adopting this calibration method, the perception performance can be significantly enhanced, providing more standardized inputs for subsequent data processing and information fusion. With a forward-looking perspective, this technique shows tremendous potential in enhancing the precision and dependability of roadside LiDAR systems, thereby fostering improved safety and efficiency across diverse applications, including autonomous vehicles, traffic monitoring, and environmental sensing. Further research and development in this direction could lead to even more sophisticated calibration techniques, ultimately optimizing the overall performance and applicability of roadside LiDAR perception systems.

## Figures and Tables

**Table 1:** Performance comparison of some roadside sensors.

Type	Advantages	Disadvantages	Max Working Distance
MMW-Radar	(a) Broad perceptual range; (b) Available for radial velocity; (c) Strong adaptability to harsh weather.	(a) Not suitable for perceiving static objects; (b) Prone to misperception.	5–200 m
Camera	(a) High resolution; (b) Available lateral velocity; (c) Excellent color perception.	(a) High computational requirements; (b) Susceptible to light interference; (c) Weather susceptible; (d) Unavailable for radial velocity.	250 m (Depending on the lens)
LiDAR	(a) Wide field of view (fov) (b) High range resolution (c) High angle resolution	(a) Inadaptability to adverse weather conditions; (b) High installation and maintenance costs.	200 m
Inductive Sensor	(a) Mature and easy to master technology; (b) Long service life; (c) High cost performance; (d) High accuracy.	(a) Difficult to install and maintain; (b) Requires road surface disruption.	100 m

**Table 2:** Euler angle computation results (partial) for Group A experiments: Euler angle calculation results for vehicle trajectories fitted with 1–30 vehicles.

The Number of Fitted Vehicles (veh)	The Calculated Results of Angle α (°)	The Calculated Results of Angle β (°)	The Calculated Results of Angle γ (°)
1	4.000000	10.000000	6.030167
2	4.000000	10.000000	5.969131
3	4.000000	10.000000	5.780432
4	4.000000	10.000000	5.779811
5	4.000000	10.000000	5.790756
6	4.000000	10.000000	5.782515
7	4.000000	10.000000	5.812183
8	4.000000	10.000000	5.861155
9	4.000000	10.000000	5.730176
10	4.000000	10.000000	5.753002
11	4.000000	10.000000	5.760653
12	4.000000	10.000000	5.774157
13	4.000000	10.000000	5.778783
14	4.000000	10.000000	5.798727
15	4.000000	10.000000	5.820700
16	4.000000	10.000000	5.846628
17	4.000000	10.000000	5.960378
18	4.000000	10.000000	5.953262
19	4.000000	10.000000	5.962495
20	4.000000	10.000000	5.968895
21	4.000000	10.000000	5.978498
22	4.000000	10.000000	5.979475
23	4.000000	10.000000	5.982899
24	4.000000	10.000000	5.988527
25	4.000000	10.000000	5.992750
26	4.000000	10.000000	5.992699
27	4.000000	10.000000	5.992969
28	4.000000	10.000000	6.002791
29	4.000000	10.000000	5.999153
30	4.000000	10.000000	5.997213
**...**	**...**	**...**	**...**

## Data Availability

The data are not publicly available due to restrictions on privacy.
